# Quality of life improvements associated with weight loss using a novel shape-shifting hydrogel capsule: RESET study results

**DOI:** 10.1038/s41366-025-01910-6

**Published:** 2025-09-16

**Authors:** Robert F. Kushner, Jamy D. Ard, Thomas A. Wadden, Patrick M. O’Neil, Harold E. Bays, Frank L. Greenway, John M. Jakicic, Holly R. Wyatt, Yael Kenan, Liora Cohen Asaraf, Donna H. Ryan

**Affiliations:** 1 Department of Medicine, Division of Endocrinology, Metabolism, and Molecular Medicine, Northwestern University Feinberg School of Medicine, Chicago, IL USA; 2 Department of Epidemiology and Prevention and Department of Medicine, Wake Forest University School of Medicine, Winston, Salem, NC USA; 3 Department of Psychiatry, Perelman School of Medicine at the University of Pennsylvania, Philadelphia, PA USA; 4 Weight Management Center, Department of Psychiatry and Behavioral Sciences, Medical University of South Carolina, Charleston, SC USA; 5 Louisville Metabolic and Atherosclerosis Research Center, Louisville, KY USA; 6 Pennington Biomedical Research Center, Louisiana State University, Baton Rouge, LA USA; 7 Division of Physical Activity and Weight Management, Department of Internal Medicine, University of Kansas Medical Center, Kansas City, KS USA; 8 Department of Nutrition Sciences, The University of Alabama at Birmingham, Birmingham, AL USA; 9 Epitomee Medical Ltd, Caesarea, Israel

**Keywords:** Weight management, Public health

## Abstract

**Objective:**

To evaluate the impact of the Epitomee capsule on quality of life (QOL) and its association with weight loss in adults with overweight and obesity.

**Methods:**

This study analyzed the RESET double-blind, placebo-controlled results, focusing on predefined QOL endpoints and post hoc analyses. A total of 279 participants with a BMI of 27–40 kg/m² were treated for 24 weeks with Epitomee or placebo, along with lifestyle intervention. Statistical analyses examined changes in IWQOL-Lite-CT scores, their relationship with weight loss, and subgroup differences.

**Results:**

Compared to the placebo group, the Epitomee group consistently showed improved outcomes in total IWQOL-Lite-CT score and Physical Function sub-score (*p* < 0.05), as well as numerically higher improvements in the Physical and Psychosocial sub-scores, although these did not reach statistical significance. Both treatment groups showed a significant improvement (*p* < 0.05) from baseline in the IWQOL-Lite-CT total score, as well as in Physical, Physical Function, and Psychosocial sub-scores over the 24-week period. A stronger correlation between weight loss and QOL improvements was observed with Epitomee vs. placebo.

**Conclusions:**

The Epitomee capsule, with lifestyle intervention, is associated with IWQOL-Lite-CT improvement, highlighting its potential as an effective non-pharmaceutical adjunct to lifestyle intervention for enhancing health outcomes in individuals with overweight and obesity.

## Introduction

Obesity, now recognized as a chronic and recurrent disease, has tripled over the last few decades, and is associated with numerous complications [1–3], including increased occurrence of multimorbidity, e.g., hypertension, cardiovascular disease, type 2 diabetes, certain cancers, obstructive sleep apnea, and musculoskeletal conditions [4, 5]. In addition, obesity has a significant impact on reducing quality of life (QOL) and is often the reason patients seek medical intervention [6]. The burden of social stigma, body-image dissatisfaction, and low self-esteem further contributes to anxiety, depression, and psychological distress associated with obesity [1, 7].

Given the numerous obesity-related complications, studies show that excess body weight significantly impairs health-related quality of life (HRQOL). The correlation between obesity severity and impairments in HRQOL is driven by functional limitations and associated weight-related medical conditions [8]. Mental health issues, including anxiety, depression, and eating disorders, can aggravate physical illnesses, further diminishing QOL [7].

There is increasing interest in incorporating patient-reported outcomes in clinical trials alongside cardiometabolic clinical endpoints as part of patient-centered care. HRQOL encompasses the effects of health on physical, mental, social, and role functioning, and can be evaluated through general-health or disease-specific measures. As a result, the Food and Drug Administration (FDA) has issued requirements for patient-reported outcome guidance [9], which are consistent with the more recent patient-focused drug-development guidance [10].

Several specialized HRQOL measures for obesity have been developed, including the widely used Impact of Weight on Quality of Life-Lite Clinical Trial (IWQOL-Lite-CT) questionnaire, which has been used to evaluate the effects of various weight-loss interventions on multiple HRQOL domains, such as overall health and physical, social, and emotional functioning [6, 11, 12].

Individuals with obesity consistently rate their general health and physical functioning lower than do individuals without obesity, with a clear gradient observed as obesity progresses from mild to severe levels, reflecting a worsening self-perceived health status [6]. The effect of obesity and mental health is bidirectional, with each condition exacerbating the other, ultimately resulting in increased mortality and reduced life expectancy [7].

The Epitomee capsule is an FDA-approved oral, non-pharmacological, non-invasive, biodegradable medical device, developed to decrease hunger and improve satiety, and resulting in weight loss while providing a favorable safety profile [13–15]. Composed of pharmaceutical-grade polymers and bonding materials, the Epitomee capsule self-expands in the stomach into a pH-sensitive gel structure, combining superabsorbent miniature polymer particles capable of absorbing water (up to 100 times their dry weight) and decreasing in volume with rising ion concentration, along with a pH-sensitive polymer envelope that is stable up to pH 6.5. Upon ingestion and water absorption, the gel particles expand, forming an elastic triangular structure that resists stomach peristaltic waves, promoting early satiety signaling. In the randomized double-blind, placebo-controlled, evaluation of efficacy and safety of the Epitomee capsule trial (RESET), the Epitomee capsule was associated with a significantly greater mean percentage of total weight loss at week 24 compared to placebo, both treatments having been combined with high-intensity lifestyle intervention (−6.6 ± 6.5% vs. −4.6 ± 4.7%; *p* < 0.0001). Of the participants who received the Epitomee capsule, 56% attained at least 5% weight loss, and 27% achieved ≥10% weight reduction compared to 11% of the placebo-treated participants [15]. Moreover, weight loss at week 24 in RESET was significantly greater in early responders to Epitomee than in early responders to placebo (−9.3 ± 6.0% vs. −6.9 ± 4.3%; *p* < 0.0001) [16]. Adverse event rates in the RESET study were similar for the Epitomee and placebo groups, and no serious device-related adverse events were observed, supporting the favorable safety profile of the Epitomee capsule [15].

In the present study, we examined predefined secondary endpoints and post-hoc analyses for treatment-related QOL changes from the RESET, measured by the IWQOL-Lite-CT at week 24 vs. baseline [15]. We sought to assess changes in treatment-related QOL, as well as association between changes in weight and QOL.

## Methods

### Study design

As described previously [15], RESET was a multicenter, pivotal, double-blind, adaptive trial conducted in the US from September 2020 through January 2023.

Following screening, 279 eligible participants were randomized in a 1:1 ratio to the Epitomee capsule plus a high-intensity lifestyle intervention, or placebo with the same lifestyle intervention for 24 weeks. Study investigators, participants, and the sponsor were blinded to the treatment group. Participants were instructed to take one Epitomee capsule or placebo capsule with at least two full cups of water (480 mL/16 oz), twice daily, 30 min before eating a main meal.

Over 24 weeks, there were 15 treatment visits: 4 in the first month, followed by bi-monthly visits. At each visit, participants had their weight and vital signs measured, received a 15 min lifestyle consultation with a registered dietitian or similar health professional [17], and were monitored for safety and capsule compliance. Laboratory tests were conducted at screening, baseline, week 12, and week 24. QOL was assessed at baseline (run-in visit) and at the end of the study (week 24) using the IWQOL-Lite-CT questionnaire.

The IWQOL-Lite-CT is a validated scale, aligned with the FDA’s patient-reported outcomes guidance recommendations. The 20-item IWQOL-Lite-CT provides a total score and three composite scores for: Physical (7 items), Physical Function (5 of the 7 Physical items), and Psychosocial (13 items) [11].

Written approval of study protocols and consent forms was obtained from the appropriate institutional review boards at each study site, and the study protocol was registered on clinicaltrials.gov (NCT04222322) [15].

### Participants

The key inclusion criteria for the RESET study were age ≥18 years with BMI ranging from 27 to 40 kg/m^2^, and either normoglycemia or prediabetes. Participants with normoglycemia were defined as having fasting plasma glucose (FPG) < 100 mg/dL and hemoglobin A1C (HbA1c) < 5.7%. Participants with prediabetes were defined as having FPG ≥ 100 mg/dL and <126 mg/dL and/or HbA1c ≥ 5.7% and ≤6.4% and were either not being treated or on a stable dose of metformin for at least 4 months prior to study entry. A maximum metformin dose of up to 2000 mg was allowed. The comprehensive list of inclusion and exclusion criteria can be found on clinicaltrials.gov (NCT04222322) [15]. All participants provided written informed consent. The study was conducted in accordance with the Declaration of Helsinki and Good Clinical Practice guidelines.

### Statistical methods

This report consists of predefined secondary endpoints and post hoc analyses. Statistical analyses were performed on observed data for participants who completed 24 weeks of treatment; participants with missing data at baseline or at week 24 were excluded from the analysis. Individual baseline scores, mean baseline scores, and mean changes from baseline to week 24 were calculated for IWQOL-Lite-CT sub-scores and total scores. IWQOL baseline scores and demographic variables were statistically compared between treatment arms. Continuous variables were analyzed with *t*-tests. When the assumption of normality was not met, the non-parametric Wilcoxon test was used for between-group comparisons. Proportions were compared using chi-square tests.

Changes in IWQOL-Lite-CT scores were also analyzed by categories of percent body weight change (less than 5%, 5 to <10%, and 10% or more) to evaluate correlations between weight-loss magnitude and changes in IWQOL-Lite-CT scores. The comparison between groups was conducted using analysis of variance on the sub-scores, with adjustments for interactions between weight-loss buckets and treatment groups. Hierarchical linear regression models were employed to determine the contribution of weight loss to changes in IWQOL-Lite-CT scores. These regression models controlled for variables that were potentially related to weight loss, including demographics (age, gender, race) baseline BMI category (27.0–29.9 ${\rm{kg}}/{{\rm{m}}}^{2}$, 30.0–34.9 ${\rm{kg}}/{{\rm{m}}}^{2}$, 35.0–39.9 ${\rm{kg}}/{{\rm{m}}}^{2}$, ≥ 40 ${\rm{kg}}/{{\rm{m}}}^{2}$*)*, baseline blood pressure (BP) category (normal BP ≤ 120/80 mmHg, abnormal BP > 120/80 mmHg), baseline glycemic status, baseline waist circumference, and IWQOL-Lite-CT scores at baseline.

Effect sizes were calculated using Cohen's d to evaluate the impact of different sub-group characteristics, including baseline BMI category, gender, and age (below or above the median age of 50 years). Participants in the overweight category (BMI: 27.0–29.9 kg/m²) and obesity Class III (BMI: ≥40 kg/m²) were excluded from the effect size analysis presented in Table 2 due to small sample size (*n* = 11 and 6, respectively). For interpretability, Cohen's d values were classified as follows: small (≈0.2), medium (≈0.5), and large (≥0.8), in line with established conventions. These classifications were used to help contextualize the practical significance of observed differences between subgroups.

All analyses were conducted using SAS and Python, employing the SciPy, Statsmodels, and Sklearn libraries for statistical calculations.

## Results

**Fig. 1:**
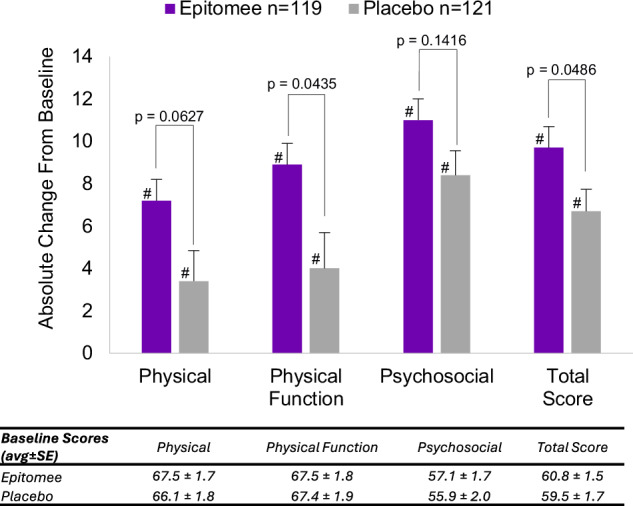
Absolute changes in IWQOL-Lite-CT scores from baseline for Epitomee (*n* = 119) and placebo (*n* = 121) treatment arms. Within-group analyses showed significantly greater changes compared to baseline for Epitomee and placebo groups (#*p* < 0.05). The Epitomee group had greater improvements in Physical Function and total IWQOL-Lite-CT scores (*p* < 0.05).

Of the 279 individuals who enrolled in the study, 240 participants (86%) completed the 24-week follow-up. Retention rates were similar across groups, with 119 of 138 participants (86.2%) completing the study in the Epitomee group and 121 of 141 participants (85.6%) in the placebo group (Consort diagram provided in Fig. 1S). As reported earlier, the proportion of participants who attained at least 10% weight loss from baseline was significantly greater in the Epitomee group than in the placebo group (27% vs. 11%; *p* < 0.002) [15].

### Absolute change from baseline in IWQOL-Lite-CT score

Baseline scores for both treatment arms were comparable, with no significant differences detected by *t*-test (Table 1S). End-of-treatment scores improved significantly from baseline across all sub-scales and the total score in both the Epitomee and placebo groups (paired *t*-test, *p* < 0.05) (Fig. 1).

Compared to the placebo group, the Epitomee group exhibited significantly greater improvements in Physical Function and IWQOL-Lite-CT total scores (Wilcoxon test, *p* < 0.05). While Physical and Psychosocial sub-scores improvements were numerically higher for the Epitomee group compared to the placebo group, but did not reach statistical significance (Fig. 1).

### Changes in IWQOL-Lite-CT score by percentage weight loss

**Fig. 2:**
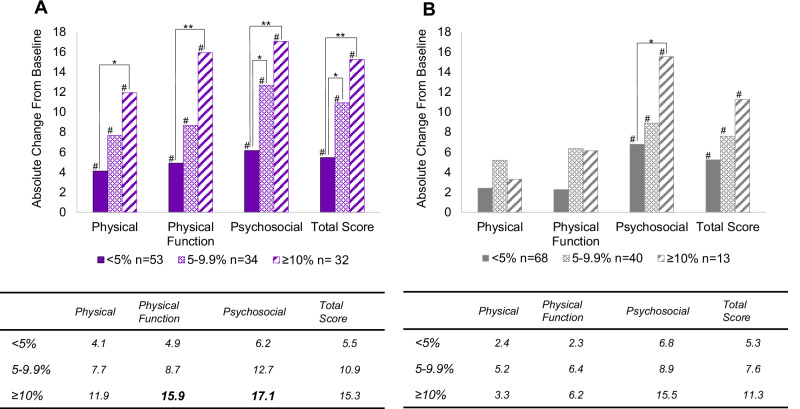
Changes in IWQOL-Lite-CT scores by percent-weight-loss categories: <5%, 5–9.9%, ≥10%. **A** Epitomee treatment arm (*n* = 119). **B** Placebo treatment arm (*n* = 121). Within-group analyses showed significantly greater changes compared to baseline for Epitomee and placebo groups (#*p* < 0.05). In the Epitomee group, all change scores were significantly smaller for participants who lost <5% of their weight compared to those who lost ≥10% (*p < 0.05, **p < 0.01), whereas in the placebo group, this was observed only for the Psychosocial score (*p < 0.05).

Changes in IWQOL-Lite-CT score by percentage weight loss groups demonstrated a stepwise trend for the Epitomee group, with increasing improvements in all IWQOL-Lite-CT scores as the percentage of weight loss increased (Fig. 2A). This trend was not observed in the placebo group, where only the Psychosocial sub-score and total IWQOL-Lite-CT score showed significant differences and improvements as the percentage of weight loss increased. Furthermore, in the Epitomee group, all changes in IWQOL-Lite-CT sub-scores and total score from baseline to week 24 in various weight-loss categories were statistically significant (paired *t*-test, *p* < 0.05), whereas only the Psychosocial sub-score and total scores were significant for the placebo group (Fig. 2B). As illustrated in Fig. 2, for the Epitomee group, all changes in sub-scores and the total score were significantly smaller for participants with <5% weight loss compared to those with ≥10% (*p* < 0.05), whereas only the Psychosocial sub-score showed significant differences in the placebo treatment group (*p* < 0.05). Psychosocial sub-score and IWQOL-Lite-CT total score were also significantly greater for Epitomee participants with greater weight loss (5–9.9%) compared to those with more modest weight loss (<5%; *p* < 0.05). No significant differences were observed in the corresponding placebo group. IWQOL-Lite-CT sub-scores and total scores did not differ between participants in the 5–9.9% vs. ≥10% weight loss categories for either the Epitomee or placebo groups. In addition, the comparison between groups revealed numerically higher improvements between the Epitomee and placebo groups in the Physical, Physical Function, Psychosocial, or total scores, although these did not reach statistical significance (*p* = 0.09, *p* = 0.06, *p* = 0.29, *p* = 0.13, respectively).

### Relationship between weight loss and IWQOL-Lite-CT total score and sub-scores

To further evaluate the relationship between weight loss and IWQOL-Lite-CT total score and sub-scores, we conducted hierarchical linear regression analyses. The visual distribution, including the estimated regression lines, is presented in Fig. 2S, and the statistical results are summarized in Table 1. The results demonstrated a positive relationship between weight loss (%) and the absolute change in IWQOL-Lite-CT total score and Physical, Physical Function, and Psychosocial sub-scores in both treatment groups (Fig. 2S). Although no significant difference was observed between the arms in a direct comparison, the Epitomee group exhibited a consistently stronger and more positive correlation between weight loss and improvements in IWQOL-Lite-CT total score and sub-scores compared to the placebo group. This is evidenced by the steeper slopes of the trend lines (Fig. 2S), higher coefficient of determination (${{\rm{R}}}^{2}$), and larger regression coefficient (*β*) observed in the Epitomee group (Table 1). This indicates that the Epitomee treatment has a more substantial positive impact on QOL compared to placebo.

**Table 1:** Effects of weight loss on changes in IWQOL-Lite-CT scores by treatment arm.

	Epitomee *n* = 119					Placebo *n* = 121				
	${{\rm{R}}}^{2\,}$	*b*	SE	*β*	*p*	${{\rm{R}}}^{2\,}$	*b*	SE	*β*	*p*
Physical	0.37	0.57	0.20	2.84	0.0054	0.35	0.28	0.29	0.97	0.3345
Physical Function	0.43	0.76	0.21	3.53	0.0006	0.33	0.41	0.34	1.20	0.2334
Psychosocial	0.49	0.87	0.17	5.01	<0.0001	0.36	0.58	0.23	2.47	0.0153
Total score	0.47	0.76	0.16	4.68	<0.0001	0.37	0.46	0.21	2.22	0.0288

Regression model included age, gender, race, body mass index (BMI), blood pressure categorization (normal BP ≤ 120/80 mmHg, abnormal BP > 120/80 mmHg), glycemic status, waist circumference at baseline, and QOL scores at baseline.

### Sub-group analysis of the effect sizes in IWQOL-Lite-CT scores for participants treated with the Epitomee capsule

Substantial improvements in IWQOL-Lite-CT scores were observed in the Epitomee treatment arm across all sub-groups, with the largest effect sizes observed in (i) participants from Obesity Class II: 35.0–39.9 ${\rm{kg}}/{{\rm{m}}}^{2}$ and (ii) participants under the age of 50 years. Notably, the effect sizes for QOL improvements were comparable between male and female participants. The most pronounced effect sizes were observed in the Psychosocial sub-score and IWQOL-Lite-CT total score among participants in the Epitomee treatment arm (Table 2).

**Table 2:** Effect sizes (Cohen’s *d*) for changes from baseline in IWQOL-Lite-CT scores for participants treated with Epitomee capsule.

	All	Baseline BMI		Gender		Age	
		Class I	Class II	Male	Female	<50	≥50
	*n* = 119	*n* = 57	*n* = 46	*n* = 23	*n* = 96	*n* = 58	*n* = 61
**Physical**	0.42	0.33	0.48	0.42	0.41	0.52	0.36
**Physical function**	0.47	0.42	0.47	0.48	0.47	0.59	0.41
**Psychosocial**	0.63	0.44	0.88	0.68	0.64	0.65	0.62
**Total score**	0.62	0.45	0.80	0.65	0.62	0.67	0.58

Participants in the Overweight category (BMI: 27.0–29.9 kg/m²) and Obesity Class III (BMI: ≥40 kg/m²) were excluded from the effect size analysis due to small sample size (*n* = 11 and 6, respectively)

## Discussion

The Epitomee group demonstrated a significantly greater improvement in total IWQOL-Lite-CT score compared to the placebo group (*p* < 0.05). In addition, improvements in Physical Function were significantly greater in the Epitomee group (*p* < 0.05), whereas the Physical and Psychosocial sub scores showed numerically higher improvements, although these did not reach statistical significance. The baseline IWQOL-Lite-CT scores in the RESET study population (e.g., Total QOL Score [mean ± SE]: 60.8 ± 1.2 in the Epitomee group and 59.5 ± 1.0 in the placebo group) are consistent with values reported in a few other obesity trials. For example, the Physical Function domain scores were comparable to those reported in the SURMOUNT-1trial [17] and the STEP 1 trial [18]. Similarly, total QOL scores at baseline were within the range reported across these studies, which span from approximately 59.5 to 65.4, depending on the population studied and trial design.

Notably, the relationship between weight change and QOL improvement was stronger among participants treated with Epitomee, as shown by linear assessments (e.g., higher ${{\rm{R}}}^{2\,}$ and β), as compared to the placebo group, based on Table 1. A clear stepwise trend was observed in the Epitomee group, demonstrating progressively significant improvements in total score and in all sub-scores of the IWQOL-Lite-CT as the percentage of weight loss increased. In contrast, for the placebo group, significant improvements with increasing weight loss were only observed in the Psychosocial sub-score. This difference may be attributed to the smaller sample size of participants achieving significant weight loss (≥10%) in the Epitomee group compared to the placebo group (27%] vs. 11%], respectively). The limited number of participants in the placebo group likely reduced the ability to detect consistent improvements across all sub-scores. This phenomenon of non-stepwise trends in a placebo group due to sample-size disparities has been observed in previous studies, such as the Liraglutide trial [19, 20]. In addition, based on Fig. 2, IWQOL-Lite-CT total score was significantly greater for Epitomee participants with greater weight loss (above 10% or 5–9.9%) compared to those with modest weight loss (below <5%). These findings align with the observation that higher weight loss generally correlates with better QOL outcomes [8, 20–22]. Clinically meaningful thresholds for within-patient improvement in the IWQOL-Lite-CT have been defined after 68 weeks of treatment with Semaglutide; as ≥13.5 points for the Physical composite, ≥14.6 for Physical Function, ≥16.2 for Psychosocial, and ≥16.6 for the Total score [12]. Although RESET study duration was shorter (24 weeks vs. 68 weeks) participants receiving Epitomee who lost >10% of their baseline weight showed clinically meaningful improvements in Physical Function (15.9) and Psychosocial (17.1) scores, exceeding these thresholds. In contrast, placebo participants with >10% weight loss did not achieve clinically meaningful improvements in any domain, including Physical Function—possibly attributed to the small sample size in the placebo subgroup (*n* = 13 in the placebo vs. 32 in Epitomee treatment arm).

Further sub-group analysis demonstrated that the effect sizes for QOL improvements, in Table 2, were consistent and meaningful across all sub scores and baseline characteristics, with Cohen’s d effect sizes ranging from 0.33 to 0.88, indicating small to large effects. These results underscore the broad and practical impact of the intervention on patient-reported outcomes across diverse subpopulations. Notably, the Psychosocial and Total score domains showed medium-to-large effects overall (Cohen's *d* = 0.63 and 0.62, respectively), indicating meaningful and clinical benefit. Participants under 50 years of age demonstrated moderately larger effect sizes across most domains compared to older participants, particularly in Physical Function (0.59 vs 0.41). In addition, participants with Obesity Class II exhibited the largest effects—particularly in the Psychosocial (0.88) and Total score (0.80), suggesting greater perceived improvements in QOL compared to those with Obesity Class I. This finding aligns with similar observations in the literature, which emphasize that the degree of obesity is strongly linked to impairments in QOL [20, 21]. As a result, individuals with more severe obesity often experience larger QOL improvements following weight loss, as their initial impairments in physical function, self-esteem, and other domains tend to be more pronounced.

Overall, the literature demonstrates a clear relationship between weight loss and QOL improvements [22–25]. The OPTIWIN program showed significant differences in Physical Function sub-score and total score at week 26, which was also demonstrated at week 52 utilizing the IWQOL-Lite scale [26]. Significant IWQOL-Lite scale improvements following 14 weeks of intensive lifestyle intervention/low-calorie diet were seen in all outcomes, except weight-related public distress. These improvements were largely maintained during 52 weeks of treatment [22]. In the SCALE study, liraglutide-treated participants had significantly greater improvements in their IWQOL-Lite total score and in all sub-scales at week 56. Notably, for both liraglutide and placebo, there was a pattern of greater improvement in IWQOL-Lite total score [19]. In both the STEP 1 and SURMOUNT-1 studies, which utilized the IWQOL-Lite-CT scale and included a non-diabetic population, similar to our study, participants experienced substantial weight loss and improvements in Physical Function sub-score. The STEP 1 trial reported a 14.9% weight loss and a 14.7-point improvement in Physical Function sub-score over 68 weeks, whereas SURMOUNT-1 showed a range of 15.0–20.9% weight loss and a range of 17.8–21.8-point improvement in Physical Function sub-score over 72 weeks [17, 18, 27]. Despite these studies being of longer duration and using a pharmacological agent, the shorter intervention in the RESET study observed an 8.9-point improvement in Physical Function sub-score, highlighting the effectiveness of the Epitomee capsule on this QOL sub-scale.

The lack of statistical significance in the linear models for the Physical and Physical Function domains in the placebo group compared to the significant improvements observed in the Epitomee group along with the overall lower gains in all IWQOL-Lite-CT scores in the placebo group, even among participants with similar weight loss, suggests that the differences observed in this study between Epitomee and placebo may be explained by factors beyond weight loss alone. The authors hypothesize that this phenomenon could be related to the Epitimee medical device’s mechanism of action or to resulting effects such as changes in hunger and satiety, especially given that all other factors were similar between the treatment groups in this double-blind, placebo-controlled study. Participants in the Epitomee group may have experienced greater consistency in satiety, along with a stronger sense of self-control and empowerment throughout the weight-loss process, as reported in a previous study with overweight or obesity and twice-daily administration of the Epitomee capsule in combination with lifestyle counseling [14], but unmeasured in this study. Further studies are needed to comprehensively explore these mechanisms.

The Epitomee capsule is a non-systemic weight loss device with a mechanical mechanism of action. Therefore, Epitomee capsule treatment may be a suitable option for individuals with overweight or obesity as an early intervention to attain moderate weight loss or for individuals with complicated medical histories and medication regimens that create numerous contraindications or potential interactions [15]. Therefore, the selection of obesity treatment is based on the risk–benefit ratio for a patient. This treatment may be particularly appropriate for individuals with overweight or obesity (BMI 25–40 kg/m²) who seek a ≥5% reduction in baseline body weight—an amount consistently associated with meaningful improvements in cardiometabolic risk factors and obesity-related conditions [28–30]. It may also serve as an alternative for patients who are either unwilling or unable to use pharmacological therapies.

The strengths of this study consist of the RESET study design, including a randomized double-blind placebo-controlled structure, the implementation of a structured lifestyle intervention in both study arms, and its conductance across multiple sites in the United States to enhance generalizability. An adequate sample size and high retention rate further ensured robust and reliable outcomes. In addition, the study utilized the validated IWQOL-Lite-CT tool.

Limitations of this study include its relatively short duration of 24 weeks and the study population, which was comprised of participants with BMI 27–40 kg/m^2,^ which limits the generalizability of the findings to individuals with more severe obesity (BMI above 40 kg/m^2^).

Another limitation is that the quality-of-life analysis was conducted on completers only. Because IWQOL-Lite-CT data were collected only at baseline and week 24, we could not apply repeated measures or imputation methods to address missing data in an intention-to-treat framework. Finally, a relatively small and imbalanced sample size by sex was analysed (18% males). While the overall sample was sufficient to detect meaningful treatment effects, this gender imbalance may limit the generalizability of findings across sexes and warrants further exploration in future studies.

Future research should aim to further explore the long-term sustainability of these weight loss-related QOL benefits and the potential impact of the Epitomee capsule on a more diverse population.

## Conclusion

In the RESET clinical trial, self-reported IWQOL-Lite-CT total outcomes significantly improved in terms of physical aspects of functioning and total QOL score in participants with overweight and obesity who were treated with Epitomee compared with placebo over 24 weeks. In post hoc analyses, improvements in QOL outcomes were numerically greater among participants achieving greater bodyweight reduction. Moreover, the strong correlation observed between weight loss and enhanced QOL underscores the Epitomee capsule’s efficacy as a non-pharmacological weight-loss treatment. When combined with lifestyle intervention, it effectively supports weight reduction while significantly improving the QOL for individuals with overweight or obesity.

## Supplementary information

Supplementary materials

## Data Availability

At this point, the dataset used in this study will not be available, as it is part of ongoing regulatory submissions in several jurisdictions.
