# Correction: Quality of life improvements associated with weight loss using a novel shape-shifting hydrogel capsule: RESET study results

**DOI:** 10.1038/s41366-025-01962-8

**Published:** 2025-11-13

**Authors:** Robert F. Kushner, Jamy D. Ard, Thomas A. Wadden, Patrick M. O’Neil, Harold E. Bays, Frank L. Greenway, John M. Jakicic, Holly R. Wyatt, Yael Kenan, Liora Cohen Asaraf, Donna H. Ryan

**Affiliations:** 1 Department of Medicine, Division of Endocrinology, Metabolism, and Molecular Medicine, Northwestern University Feinberg School of Medicine, Chicago, IL USA; 2 Department of Epidemiology and Prevention and Department of Medicine, Wake Forest University School of Medicine, Winston, Salem, NC USA; 3 Department of Psychiatry, Perelman School of Medicine at the University of Pennsylvania, Philadelphia, PA USA; 4 Weight Management Center, Department of Psychiatry and Behavioral Sciences, Medical University of South Carolina, Charleston, SC USA; 5 Louisville Metabolic and Atherosclerosis Research Center, Louisville, KY USA; 6 Pennington Biomedical Research Center, Louisiana State University, Baton Rouge, LA USA; 7 Division of Physical Activity and Weight Management, Department of Internal Medicine, University of Kansas Medical Center, Kansas City, KS USA; 8 Department of Nutrition Sciences, The University of Alabama at Birmingham, Birmingham, AL USA; 9 Epitomee Medical Ltd, Caesarea, Israel

**Keywords:** Weight management, Public health

Correction to: *International Journal of Obesity* 10.1038/s41366-025-01910-6, published online 16 September 2025

In this article, the author’s name Donna H. Ryan was incorrectly given as Ryan H. Ryan.

The original article has been corrected.

The original article can be found online at 10.1038/s41366-025-01910-6

