# Supplementary material for: Quality of life improvements associated with weight loss using a novel shape-shifting hydrogel capsule: RESET study results
Source: Int J Obes (Lond). 2025 Sep 16;50(1):159–65. doi: 10.1038/s41366-025-01910-6 (PMC12855005; doi:10.1038/s41366-025-01910-6)
Supplement: Supplementary file 1 — Supplementary materials [file 41366_2025_1910_MOESM1_ESM.pdf]

Supplementary Information

Quality of Life Improvements Associated with Weight Loss Using a Novel Shape-Shifting Hydrogel Capsule: RESET Study Results

AUTHORS:

Robert F. Kushner[1], Jamy D. Ard[2], Thomas A. Wadden[3], Patrick M. O'Neil[4], Harold E. Bays[5], Frank L. Greenway[6], John M. Jakicic[7], Holly R. Wyatt[8], Yael Kenan[9], Liora C. Asaraf[9] and Donna H. Ryan[6]

AFFILIATIONS:

[1]Department of Medicine, Division of Endocrinology, Metabolism, and Molecular Medicine, Northwestern University Feinberg School of Medicine, Chicago, Illinois, USA

[2]Department of Epidemiology and Prevention and Department of Medicine, Wake Forest University School of Medicine, Winston-Salem, North Carolina, USA

[3]Department of Psychiatry, Perelman School of Medicine at the University of Pennsylvania, Philadelphia, Pennsylvania, USA

[4]Weight Management Center, Department of Psychiatry and Behavioral Sciences, Medical University of South Carolina, Charleston, South Carolina, USA

[5]Louisville Metabolic and Atherosclerosis Research Center, Louisville, Kentucky, USA

[6]Pennington Biomedical Research Center, Louisiana State University, Baton Rouge, Louisiana, USA

[7]Division of Physical Activity and Weight Management, Department of Internal Medicine, University of Kansas Medical Center, Kansas City, Kansas, USA

[8]Department of Nutrition Sciences, The University of Alabama at Birmingham, Birmingham, Alabama, USA

[9]Epitomee Medical Ltd., Caesarea, Israel

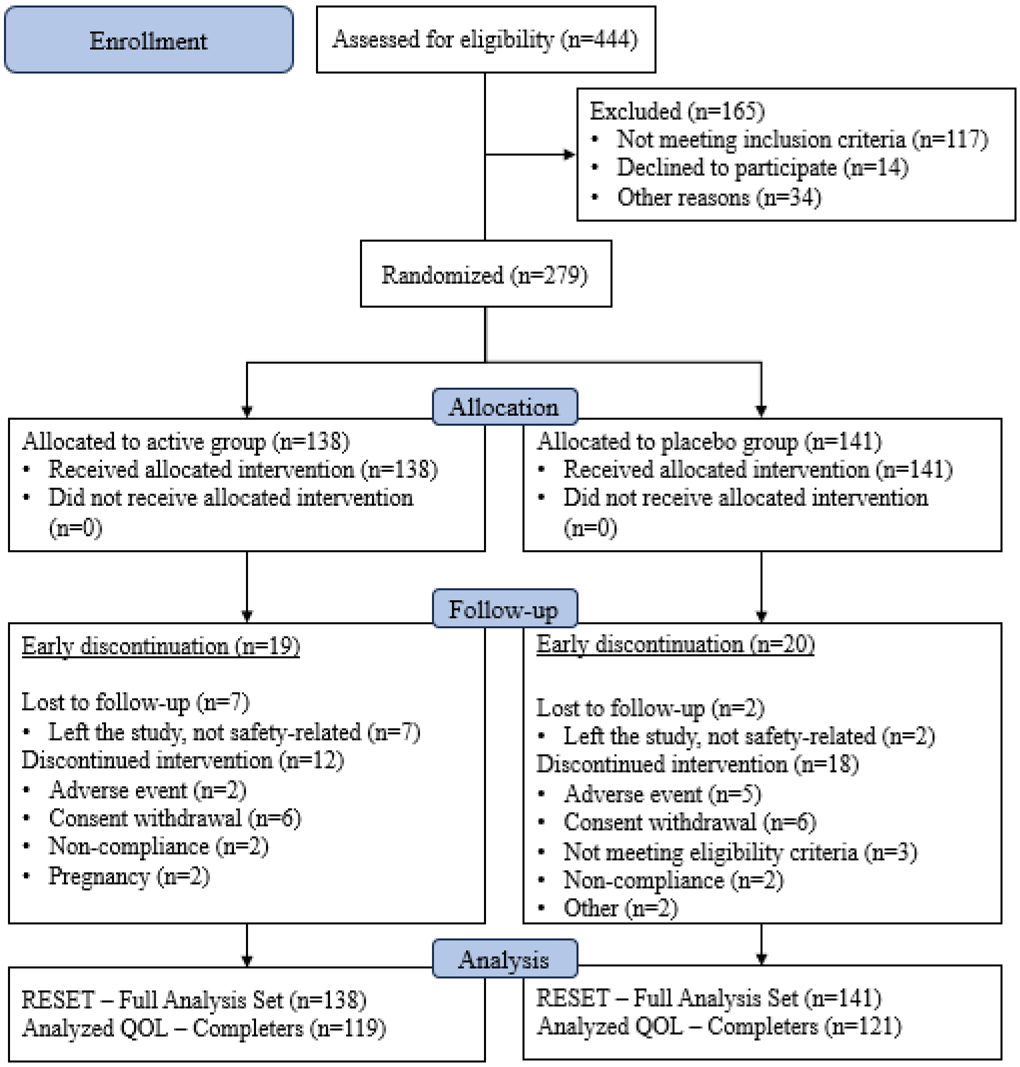

Figure 1S: CONSORT (Consolidated Standards of Reporting Trials) Flow Diagram

Table 1S - Demographics and baseline characteristics

	Epitomee n=119	Placebo n=121	P-value
Gender %(n/N)			0.6024
Female	80.7 (96/119)	76.9 (93/121)	
Male	19.3 (23/119)	23.1 (28/121)	
Ethnicity %(n/N)			0.5525
Caucasian	73.1 (87/119)	65.3 (79/121)	
Black or African-American	19.3 (23/119)	23.1 (28/121)	
Others	7.6 (9/119)	11.6 (14/121)	
Age, years, mean [SD]	49.5 [12.6]	48.9 [12.3]	0.7042
Weight, kg, mean [SD]	96.4 [16.1]	95.8 [16.1]	0.7849
BMI, kg/m^2, mean [SD]	34.1 [3.3]	33.7 [3.4]	0.3291
Weight categories, % (n/N)			0.1772
Overweight (BMI, 25.0–29.9 kg/m2)	9.2 (11/119)	17.4 (21/121)	
Class I (BMI, 30.0–34.9 kg/m2)	47.9 (57/119)	43.8 (53/121)	
Class II (BMI, 35.0–39.9 kg/m2)	38.7 (46/119)	38 (46/121)	
Class III (BMI ≥ 40.0 kg/m2)[a]	4.2 (5/119)	0.8 (1/121)	
Glycemic status, % (n/N)			0.4732
Missing	1.7 (2/119)	0.0 (0/121)	
Normoglycemic	57.1 (11/119)	64.5 (78/121)	
Prediabetic	38.7 (47/119)	34.7 (42/121)	
Diabetes [b]	2.5 (3/119)	0.8 (1/121)	
IWQOL-Lite CT mean [SD]			
Physical	67.5 [18.0]	66.1 [19.6]	0.5549
Physical Function	67.5 [20.1]	67.4 [20.5]	0.9621
Psychosocial	57.1 [18.3]	55.9 [21.8]	0.6429
Total	60.8 [16.2]	59.5 [19.0]	0.5744

[a] 3 ineligible participants with BMI higher than 40 were included by mistake due to the slight weight change between screening and randomization. Study's medical monitor and the responsible principal investigators (PIs) decided to allow their continued participation

[b] 4 ineligible participants with diabetes included by mistake. Study's medical monitor and the responsible principal investigators (PIs) decided to allow their continued participation

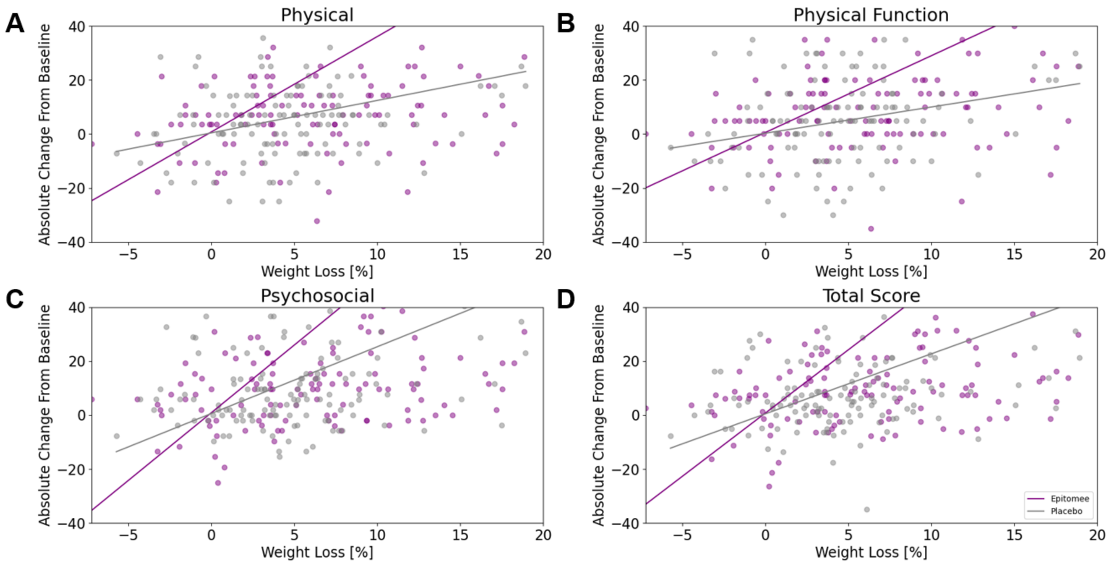

Figure 2S: Relationship between percentage of weight loss and change in IWQOL-Lite-CT composite scores from baseline to end of study with Epitomee capsule vs. placebo. The purple dots and line represent Epitomee Capsule, and the grey dots and line represent placebo. **A.** Relationship between % weight loss and Physical score. **B.** Relationship between weight loss and Physical Function score. **C.** Relationship between weight loss and Psychosocial score. **D.** Relationship between weight loss and Total score.
